# Efficacy and mechanism of acupuncture for ischemic poststroke depression: Study protocol for a multicenter single-blinded randomized sham-controlled trial

**DOI:** 10.1097/MD.0000000000014479

**Published:** 2019-02-15

**Authors:** Hai Lu, Menghan Li, Bo Zhang, Xuesong Ren, Lina Meng, Weijing Bai, Li Wang, Zhenzhen Wang, Shaojie Ding, Yuanyuan Gan, Zhilong Zhang, Peifang Li, Linpeng Wang, Zhihong Meng, Hong Zhao, Fei Wang, Chunhong Zhang

**Affiliations:** a First Teaching Hospital of Tianjin University of Traditional Chinese Medicine; b Tianjin University of Traditional Chinese Medicine; c Tianjin Academy of Traditional Chinese Medicine Affiliated Hospital, Tianjin; d Second Affiliated Hospital of Anhui University of Traditional Chinese Medicine, Hefei; e Beijing Hospital of Traditional Chinese Medicine of Capital Medical University, Beijing, China.

**Keywords:** ^1^H-MRS, acupuncture, fluoxetine hydrochloride, HAMD-17, post-stroke depression, RCT, SDS, sham-acupionts, study protocol

## Abstract

**Introduction:**

Poststroke depression is a serious and common complication of stroke, especially the ischemic poststroke depression. Antidepressants are used in poststroke depression, and acupuncture may be an alternative approach. However, the efficacy and mechanism of acupuncture for poststroke depression has not been confirmed.

**Methods/design:**

This is a multicenter, central-randomized, single-blind, sham-controlled clinical trial. We will allocate 208 subjects aged between 40 and 80 years old, diagnosed with initial poststroke depression (PSD) within 6 months to 2 groups randomly in a ratio of 1:1. Patients in the experimental group will be treated with traditional acupuncture and placebo pills, whereas the others in the control group will be treated with sham-acupoints acupuncture and antidepressant (fluoxetine hydrochloride tablets). All will be given acupuncture and/or medication treatment for 12 weeks, and then received 12-week follow-up. Patients will be evaluated with the 17-item Hamilton Depression Scale and Se1f-rating Depression Scale for depression state, National Institute of Health Stroke Scale for neurological deficit, Modified Barthel Index for activities of daily living, Treatment Emergent Symptom Scale for side effects of treatments, diagnosis and evaluation criteria of traditional Chinese medicine for stroke (try out) for curative effects of stroke, and clinical global impression for synthesize effect before and the 2nd, 4th, 8th, and 12th week of treatment, 24th week of follow-up. Study on mechanisms of acupuncture will be revealed through the diversity of brain metabolites (choline-containing compounds [Cho], N-acetylaspartate [NAA], myoinositol, glutamine and glutamate complex, creatine [Cr], Cho/Cr, Cho/NAA, Cr/NAA) in bilateral dorsolateral prefrontal cortex and anterior cingulate cortex monitored by proton magnetic resonance spectroscopy, and serum monoamine neurotransmitters (5-hydroxytryptamine, norepinephrine, dopamine) and cytokines (brain-derived neurotrophic factor [BDNF], interleukin [IL]-4, IL-6, IL-10, IL-18, IL-1β, tumor necrosis factor alpha) before and the 12th week of treatment. Baseline characteristics of patients will be summarized by groups and compared with chi-square for categorical variables, and 2-sample *t* tests or Wilcoxon rank-sum test for the continuous variables. Primary and secondary outcomes according to the measurement times are applicable to univariate repetitive measurement deviation analysis or 2-sample *t* tests, or Wilcoxon rank-sum test.

**Conclusion:**

The present research is designed to investigate efficacy and mechanism of traditional acupuncture therapy on ischemic PSD, also to explore the correlation between cerebra metabolic and serologic factors, and ischemic PSD. With this research, we are looking forward to find out an appropriate alternative nondrug therapy for PSD people to alleviate the adverse effects and drug dependence caused by antidepressants.

## 1 Introduction

Poststroke depression (PSD) is a serious and common complication of stroke.^[1]^ Reports revealed that the prevalence of PSD was from 30% to 65%.^[2,3]^ PSD has been associated negatively with survival, cost of medical care, and compliance with therapy including rehabilitation, functional outcome, resumption of social activities, and quality of life.^[4]^

At present, the treatment of PSD mostly applies selective serotonin reuptake inhibitors (SSRIs). A meta-analysis of randomized controlled trials for PSD has revealed that patients in the SSRIs group had make great improvement in depressive symptoms significantly compared with the placebo group.^[5]^ Several controlled studies have demonstrated that while some patients made improvement on depression scores significantly, others discontinued due to side effects.^[6,7]^ And SSRIs have slow onset speed about 3 to 4weeks.^[5]^ A trial suggested that the advantages of fluoxetine hydrochloride could not be demonstrated within the first 3 months compared with controlled treatment.^[8]^ On the contrary, the use of antidepressants might also increase the risk of interactions derived from various drugs, as most stroke patients concurrently take.^[9]^

Acupuncture is 1 of the main treatments of traditional Chinese medicine (TCM) which has been used for over 2000 years. Acupuncture is widely used in the treatment of PSD. A meta-analysis has demonstrated that the curative effect of acupuncture on PSD was superior to antidepressants among waitlist controls in improving both response and symptom severity.^[10]^ Another meta-analysis revealed that there is no difference in effective rate on comparison between the acupuncture group and western medicine group in treating PSD.^[11]^

In China, because of the good effect, more and more patients are willing to choose acupuncture for PSD. However, no sufficient clinical trial data and solid evidence could confirm whether acupuncture could yield certain efficiency in treating PSD and how acupuncture works. It is necessary to verify the effectiveness and mechanism of acupuncture from senior quality studies. Now, the evidence about acupuncture effect on treating PSD is inadequate and must apply further strictly design clinical studies.

The primary objectives of this study are to evaluate the efficacy and mechanism of acupuncture treatment and follow-up on PSD during 6 months.

## 2 Methods/design

### 2.1 Study design

This is a multicenter, central-randomized, single-blind, sham-controlled, noninferiority clinical trial. In all, 208 participants will be randomized into 2 groups at a ratio of 1:1. Participants in the treatment group will be treated with traditional acupuncture and placebo medicine. Participants in the control group will be treated with sham-acupoint acupuncture and medicine (fluoxetine hydrochloride tablets).

**Figure 1:**
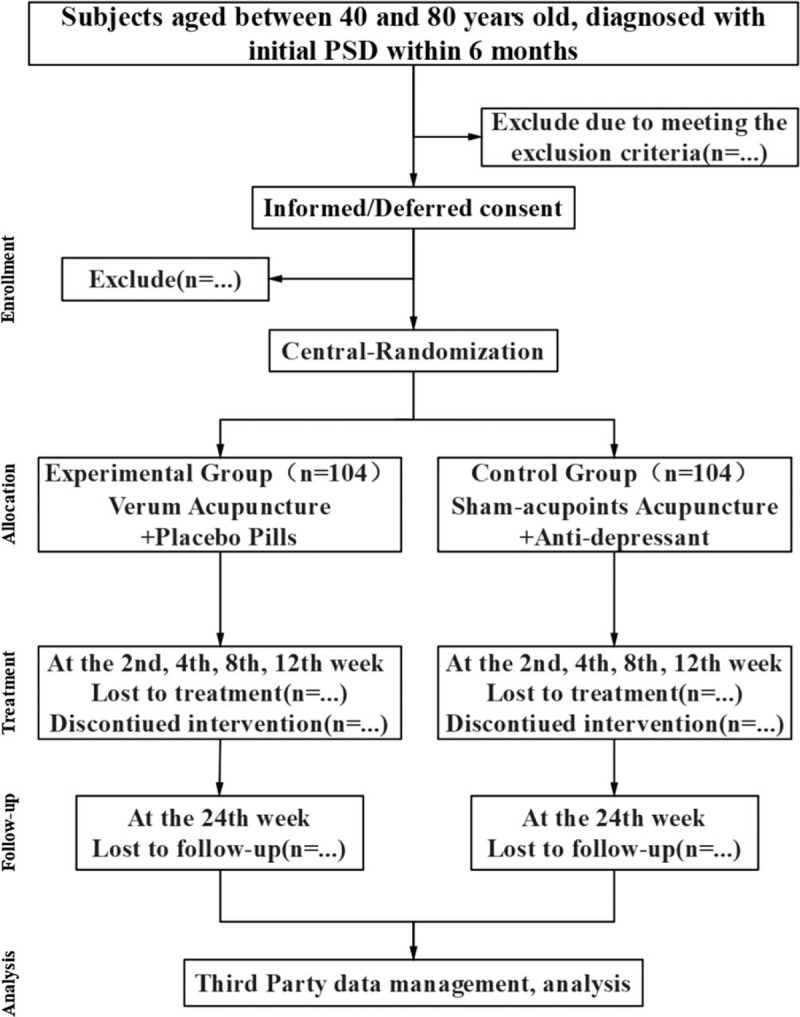
Study design and participant flow chart.

The whole study period is 25 weeks, including 1-week baseline observation, 12-week treatment, and 12-week follow-up. Patients will be evaluated with scales for clinical efficacy before and the 2nd, 4th, 8th, 12th week of treatment, and 24th week of follow-up, and monitored by proton magnetic resonance spectroscopy (^1^H-MRS) and serum for acupuncture mechanisms before and the 12th week of treatment (Fig. 1).

The clinical trial results will be reported according to the Standards for Reporting Interventions in Clinical Trials of Acupuncture (STRICTA) statement.^[12]^

### 2.2 Ethics

This study has been approved by the ethics committee of First Teaching Hospital of Tianjin University of Traditional Chinese Medicine, and registered on *ClinicalTrials.gov* with the ID NCT02472613. Informed consents will be obtained from all the subjects involved.

### 2.3 Study population

#### 2.3.1 Inclusion criteria

The inclusion criteria are as follows:

1. Subjects diagnosed with ischemic stroke in International Classification of Diseases (ICD-10-I63.902).^[13]^
2. Subjects diagnosed with secondary mild and moderate-grade depression, 17-item Hamilton Depression Scale (HAMD-17) scores between 7 and 24 points^[14]^, according to the Diagnostic and Statistical Manual of Mental Disorders-Fourth Edition (DSM-IV) or Chinese Classification and Diagnostic Criteria of Mental Disorders-Third Edition (CCMD-III).^[15,16]^
3. Subjects aged between 40 and 80 years old, male or female.
4. Subjects diagnosed with depression for the first time after ischemic stroke.
5. Subjects diagnosed with initial PSD within 6 months.
6. Cooperation during examination, without aphasia and severe cognitive disorders, Mini-Mental State Examination (MMSE) scores >17 points^[17]^.

#### 2.3.2 Exclusion criteria

The exclusion criteria are as follows:

1. Subjects diagnosed with primary depression or secondary major depressive disorder, HAMD-17 scores >24 points^[14]^.
2. Participation in any other clinical trial or taking antidepressants in recent 2 weeks.
3. With aphasia and severe cognitive dysfunction (MMSE scores <17 points).^[17]^
4. With severe heart disease, malignancy, kidney, and liver function insufficiency.
5. Pregnant or lactating women.

### 2.4 Study settings and recruitments

The present study will be conducted in the following 3 hospitals: First Teaching Hospital of Tianjin University of Traditional Chinese Medicine, Beijing Hospital of Traditional Chinese Medicine of Capital Medical University, Tianjin Academy of Traditional Chinese Medicine Affiliated Hospital, and Second Affiliated Hospital of Anhui University of Traditional Chinese Medicine (later joined).

Participants with the diagnosis of ischemic stroke with depression will be recruited at the above mentioned 4 hospitals, who mainly come from hospital wards and clinics. Apart from this, posters, leaflets, and the routine-free clinics will also be helpful. Sure, except these, the hospital websites, WeChat, and Microblogging are powerful advocacy media.

### 2.5 Study group

In all, 208 subjects who meet the criteria with informed consents will be selected. The subjects will be randomly divided into 2 groups, namely the experimental group and control group, 104 cases in each group.

### 2.6 Study time

This clinical study was conducted from May, 2016 to June, 2018. It is expected that all subjects’ follow-up observation will end in the second half of 2019 because of the delayed recruitment.

### 2.7 Interventions

Interventions were selected based on the theory of TCM and expert-level discussions. The acupuncturists involved have received their practitioners’ license from China's National Health and Family Planning Commission, who have worked for more than eight years. Subjects in experimental group received verum acunpuncture and placebo pills, and others in control group received sham-acupionts acunpuncture and medications. Apart from this, the regular drugs used in neurological department are allowed, such as antihypertensive drugs, lipid-regulating drugs, and antiplatelet drugs.

#### 2.7.1 Experimental group

##### 2.7.1.1 Verum acupuncture

Disposable sterile acupuncture needles (0.25 × 40 mm; Hwato Brand, Suzhou Medical Supplies Factory Co., Ltd., Suzhou, China) were used. “*Tiaoshen-Kaiqiao*” acupuncture is applied to treat PSD according to TCM theory. Acupoints are including bilateral PC6 (*Neiguan*), GV26 (*Shuigou*), EX-HN3 (*Yintang*), GV23 (*Shangxing*), GV20 (*Baihui*), EX-HN1 (*Sishencong*), bilateral ST2 (*Sibai*), bilateral GB20 (*Fengchi*), and affected side LI15 (*Jianyu*), LI11 (*Quchi*), LI10 (*Shousanli*), LI4 (*Hegu*), GB31 (*Fengshi*), SP10 (*Xuehai*), ST36 (*Zusanli*), SP6 (*Sanyinjiao*) and LR3 (*Taichong*).

Acupoints GV26, GV23, GV20, ST2, and EX-HN1 are inserted at the depth of 5 to 10 mm, whereas others are inserted at the depth of 20 to 30 mm. Then pecking-acupuncture technique is done on GV26 to achieve strong stimulation for 6 to 9 seconds. Lifting-thrusting and twirling-rotating manipulations are conducted on others for called “*De Qi*” sensation, like the feeling of soreness, numbness, heaviness, and distension. Finally, all needles would be removed after retained for 30 minutes. Participants will be treated 3 sessions per week for 12 consecutive weeks.

##### 2.7.1.2 Placebo pills

Ingredients of the placebo pills are starch produced by TIPR Pharmaceutical Co., Ltd, which exactly have the same shape and taste as fluoxetine hydrochloride tablets. Two tablets each time, one time a day for 12 weeks.

#### 2.7.2 Control group

##### 2.7.2.1 Sham-acupoints acupuncture

Sham-acupoints acupuncture mainly consists of some sham-acupoints, which have no therapeutic effect on depression in light of literatures^[18]^ and clinical experience, such as SJ14 (*Jianliao*), PC2 (*Tianquan*), LU4 (*Xiabai*), SJ12 (*Xiaoluo*), LI13 (*Shouwuli*), SJ9 (*Sidu*), SJ7 (*Huizong*), LI7 (*Wenliu*), LI12 (*Zhouliao*), PC3 (*Quze*), LI6 (*Kongzui*), SJ6 (*Zhigou*), SJ3 (*Zhongzhu*), ST32 (*Futu*), SP11 (*Jimen*), ST33 (*Yinshi*), ST34 (*Liangqiu*), ST37 (*Shangjuxu*), ST39 (*Xiajuxu*), GB33 (*Xiyangguan*), LR5 (*Ligou*), BL61 (*Pucan*), BL64 (*Jinggu*), KI2 (*Rangu*), LR5 (*Ligou*), ST44 (*Neiting*) on the affected side upper and lower extremities. Manipulators should choose aboved acupionts in accordance with verum acupuncture group about the number of acupoints and the stimulating amount of acupuncture, whereas, without “*De Qi*” sensation. The rest are the same as the verum acupuncture group.

##### 2.7.2.2 Fluoxetine hydrochloride

Fluoxetine hydrochloride tablets (10 mg per tablet; Changzhou Siyao Pharmaceutical Co., Ltd., Changzhou, China) as a SSRI are used. Two tablets each time, one time a day for 12 weeks.

### 2.8 Adverse events observation

It could be that patients with subcutaneous bleeding or hematoma, needle sickness, and pain around acupionts at needling are more likely related to acupuncture. Sickness, vomiting, diarrhea, headache, anxiety, insomnia, drowsiness, fatigue, sweating, tremors, and skin rash occur in patients are probably connected to drugs. With the Treatment Emergent Symptom Scale (TESS) and patients’ diary, all adverse events are to be recorded and measured during treatment and the follow-up period. Serious adverse events should be reported to the principal investigator immediately. All details will be documented, appropriate medical care will be given at once.

### 2.9 Primary outcomes

The HAMD-17 and Se1f-rating Depression Scale (SDS)^[19]^ scores for depression state before and the 2nd, 4th, 8th, 12th week of treatment, and 24th week of follow-up. The diversity of choline-containing compounds (Cho), N-acetylaspartate (NAA), myoinositol (MI), glutamine and glutamate complex (Glx), creatine (Cr), Cho/Cr, Cho/NAA, and Cr/NAA in bilateral dorsolateral prefrontal cortex and anterior cingulate cortex. The levels of 5-hydroxytryptamine (5-HT), norepinephrine (NE), dopamine (DA), brain-derived neurotrophic factor (BDNF), intereukin (IL)-4, IL-6, IL-10, IL-18, IL-1β, and tumor necrosis factor alpha (TNF-α) in serum before and the 12th week of treatment.

### 2.10 Secondary outcomes

National Institute of Health Stroke Scale (NIHSS)^[20]^ scores for neurological deficit, Modified Barthel Index (MBI)^[21]^ scores for activities of daily living (ADL), TESS^[22]^ scores for side effects of treatments, diagnosis and evaluation criteria of TCM for stroke (try out)^[23]^ scores for curative effects of stroke, and clinical global impression (CGI)^[24]^ scores for synthesize effect before and the 2nd, 4th, 8th, 12th week of treatment, and 24th week of follow-up. Patients’ diary for tracking their mood, sleep, diet, medications, adverse events, and others during the research was maintained. All the dropouts and causes will be documented in case report form (CRF).

### 2.11 Estimation of sample size

Based on the previous pilot study,^[25]^ the mean change in HAMD-17 before and after treatment was used as the indicator for the efficacy evaluation in the calculation of the sample size. Results from our previous studies showed that the mean HAMD-17 scores change was 10.49 ± 4.55 after acupuncture treatment and 10.24 ± 4.46 after fluoxetine treatment. Thus, we performed a noninferiority test to calculate the appropriate trial sample size with 90% power, beta of 0.1, alpha of 0.05. The results showed that a clinically important difference can be detected by a sample size of at least 84 in each group. This number was then increased to 104 per group to allow a predicted 20% dropout rate. We will plan to enroll in all 208 participants in this study.

### 2.12 Randomization and blinding

After recruitment, the Central Randomization System performed by the China Academy of Chinese Medical Sciences (CACMS) would be used to randomize patients who meet the eligibility criteria into 2 groups at a ratio of 1:1. Every center will compete for the random numbers from random sequence generated by the Central Randomization System. Each subject will be registered with a unique ID. After the clinical screening and getting the informed consent forms, subjects will be assigned to experimental group (verum acupuncture + placebo pills) and control group (sham-acupoints acupuncture + fluoxetine) randomly. Each subject will receive the similar the number of acupoints and medications, as well as the stimulating amount of needling. Both groups will be in the package with the same looking and similar inner feeling, whereas all subjects will not be aware of the grouping.

### 2.13 Statistical analysis

Data analysis will be based on intention-to-treat analysis (ITT) and/or per-protocol analysis (PP) principle in the light of missing data. We will also evaluate the group effect by comparing the analysis results between the above 2 data sets to evaluate robustness of our analytical results.

All data will be analyzed using SPSS19.0 (v19.0, SPSS Inc., Chicago, IL) in the China Academy of Chinese Medical Sciences (CACMS). A *P* value <.05 will be considered as significant. Descriptive statistics will be performed with mean, standard deviation (SD), maximum, minimum, and so on by groups and time.

Baseline characteristics of patients will be summarized by groups and compared with chi-square for categorical variables, and 2-sample *t* tests (normal distributions) or Wilcoxon rank-sum test (non-normal distributions) for the continuous variables. Primary and secondary outcomes according to the measurement times are applicable to univariate repetitive measurement deviation analysis or 2-sample *t* tests, or Wilcoxon rank-sum test. If there is a violation of distribution assumption, appropriate transformation will be used.

#### 2.13.1 Primary outcomes

We will use univariate repetitive measurement deviation analysis with HAMD-17 and SDS scores before and the 2nd, 4th, 8th, 12th week of treatment, and 24th week of follow-up as the outcomes. A significant effect of group indicates that the HAMD-17 and SDS scores are different between groups after interventions.

The diversity of Cho, NAA, MI, Glx, Cr, Cho/Cr, Cho/NAA, and Cr/NAA will be compared with 2-sample *t* tests or Wilcoxon rank-sum test with their concentrations or ratios before and the 12th week of treatment as the outcome variables. Similar analysis approach will be used for the levels of 5-HT, NE, DA, BDNF, IL-4, IL-6, IL-10, IL-18, IL-1β, and TNF-α in serum.

#### 2.13.2 Secondary outcomes

Continuous outcome variables including NIHSS scores, MBI scores, diagnosis and evaluation criteria of TCM for stroke (try out) scores and CGI scores before and the 2nd, 4th, 8th, 12th week of treatment, and 24th week of follow-up will be analyzed with univariate repetitive measurement deviation analysis. Categorical outcome variables in the patients’ diary and chi-square test will be used for comparison between groups.

#### 2.13.3 Safety evaluation

Adverse effects will be evaluated by TESS scores with univariate repetitive measurement deviation analysis between groups at different time. Adverse events will be analyzed as a multiply variable. The number and percentage of patients with AE will be calculated and compared using chi-square test.

### 2.14 Quality control

The reasons of dropouts or withdrawals throughout the treatment and follow-up periods will be fully recorded. To ensure trial quality, the quality monitors will verify all the process details at regular intervals and check the authenticity of the data. Moreover, a third party (CACMS) will be invited to manage the data independently.

Because differences among centers can cause bias, to reduce the bias, the evaluation of the rating scales in every subcenter will be executed by 2 psychiatrists who have been trained with the same criterion of evaluating. Also, there are 6 psychiatrists in total. All acupuncturists will receive special training about the study and manipulations including locations of acupoints, depth, and direction of needling, frequency of lifting and inserting, and so on before recruitment. To record the attendance and compliance, we make record cards for patients which cover date of treatment, personal information, and their signatures after every treatment.

## 3 Discussion

Poststroke depression is a secondary neuropsychiatry disease, which is now considered its pathogenesis involving endogenous factors like neurochemical substances and inflammatory cytokines, and also exogenous factors like sex, age, marriage, social environment, education, life stress, and so on by reference to the primary depression.^[26]^ It is still unknown whether the pathogenesis is with particularity for PSD itself. Apart from this, the PSD diagnosis is confusion. The CCMD-III, ICD-10, and DSM-IV are the most commonly used in China.^[27]^ However, there was no clear diagnostic criterion for PSD above. DSM-IV categorizes PSD as “Mood disorder due to a general medical condition (ie, stroke),” with the specificities of depressive features, major depressive-like episodes, manic features, or mixed features. Refer to the diagnostic criteria of depressive episode in CCMD-III, ischemic stroke patients who were in depressed mood will be diagnosed with PSD. Therefore, according to patient symptoms combined with rating scale scores, we hope to obtain high-quality research data from this multicenter clinical trial that could provide reliable basis to future related study.

Fluoxetine hydrochloride, which is chosen as a standard drug therapy in this study, has been found to be effective and safe in PSD.^[28,29]^ A large number of ischemic stroke patients are recommended to take clopidogrel to prevent thrombosis.^[30]^ However, antidepressants might also increase the risk of drug-drug interactions. For example, SSRIs like fluoxetine and fluvoxamine are always avoided in ischemic heart disease patients who are receiving clopidogrel.^[31]^ Also, other studies have found that fluoxetine may potentially diminish the efficacy of clopidogrel.^[32]^ Moreover, the US Food and Drug Administration (FDA) reminded the public to avoid fluoxetine hydrochloride combination with clopidogrel because of the reasons above,^[33]^ especially for stroke patients. Hence, patients diagnosed with depression after ischemic stroke who were useing clopidogrel would not be recommended to take fluoxetine.^[34]^ For this, acupuncture on PSD may be a promising alternative therapy.

Acupuncture has been widely used and received in clinical practice in China.^[35]^ Patients in China are usually familiar with acupuncture, and expect to receive acupuncture treatments when they participate in this kind trial. Apart from this, because acupuncture is often regarded as an invasive operation,^[36]^ it is difficult to design a nonpenetrating sham acupuncture, which does not produce any physiological effects,^[37]^ and would not be experienced and recognized by patients at home. However, to improve subject compliance and achieve blinding, sham acupuncture will be necessary in the control group, whereas placebo pills will be adopted in experimental group in a high-quality RCT. So, in this study, sham acupuncture and placebo pills are applied simultaneously to eliminate the placebo effect. We choose needling at blunt acupoints as sham acupuncture without effect on depression, based on literatures^[18]^ and clinical experience.

Although studies have showed the efficacy of acupuncture for PSD,^[38,39]^ multicenter and large-sample RCTs are still lacking, and also the relevant mechanism research, which would be helpful to provide high-quality evidence for evaluation of acupuncture on PSD. Our protocol was designed through an extensive discussion by reference to several previous related studies.^[40–42]^ We try our best to attempt to minimize the biases that may influence study results. Finally, we intend to demonstrate the hypothesis that acupuncture as an alternative may be validated for PSD.

## Author contributions

CHZ and ZHM designed this study together. HL, MHL and BZ drafted the protocol. FW performed the statistical analysis. CHZ and ZHM was responsible for the writing revision. All authors read and approved the final manuscript.

**Conceptualization:** Zhihong Meng, Chunhong Zhang.

**Data curation:** Xuesong Ren, Lina Meng, Weijing Bai, Li Wang, Zhenzhen Wang, Shaojie Ding, Yuanyuan Gan.

**Formal analysis:** Fei Wang.

**Funding acquisition:** Chunhong Zhang.

**Investigation:** Zhihong Meng.

**Methodology:** Hong Zhao, Chunhong Zhang.

**Project administration:** Zhilong Zhang, Peifang Li, Linpeng Wang, Chunhong Zhang.

**Software:** Fei Wang.

**Supervision:** Chunhong Zhang.

**Writing – original draft:** Hai Lu, Menghan Li, Bo Zhang.

**Writing – review & editing:** Zhihong Meng, Chunhong Zhang.
